# Supplementary material for: Discovery of epigenetically silenced tumour suppressor genes in aggressive breast cancer through a computational approach
Source: NAR Cancer. 2025 Jun 18;7(2):zcaf020. doi: 10.1093/narcan/zcaf020 (PMC12203794; doi:10.1093/narcan/zcaf020)
Supplement: zcaf020_Supplemental_Files [file zcaf020_supplemental_files.zip › Supplementary_Figure_S1.pdf]

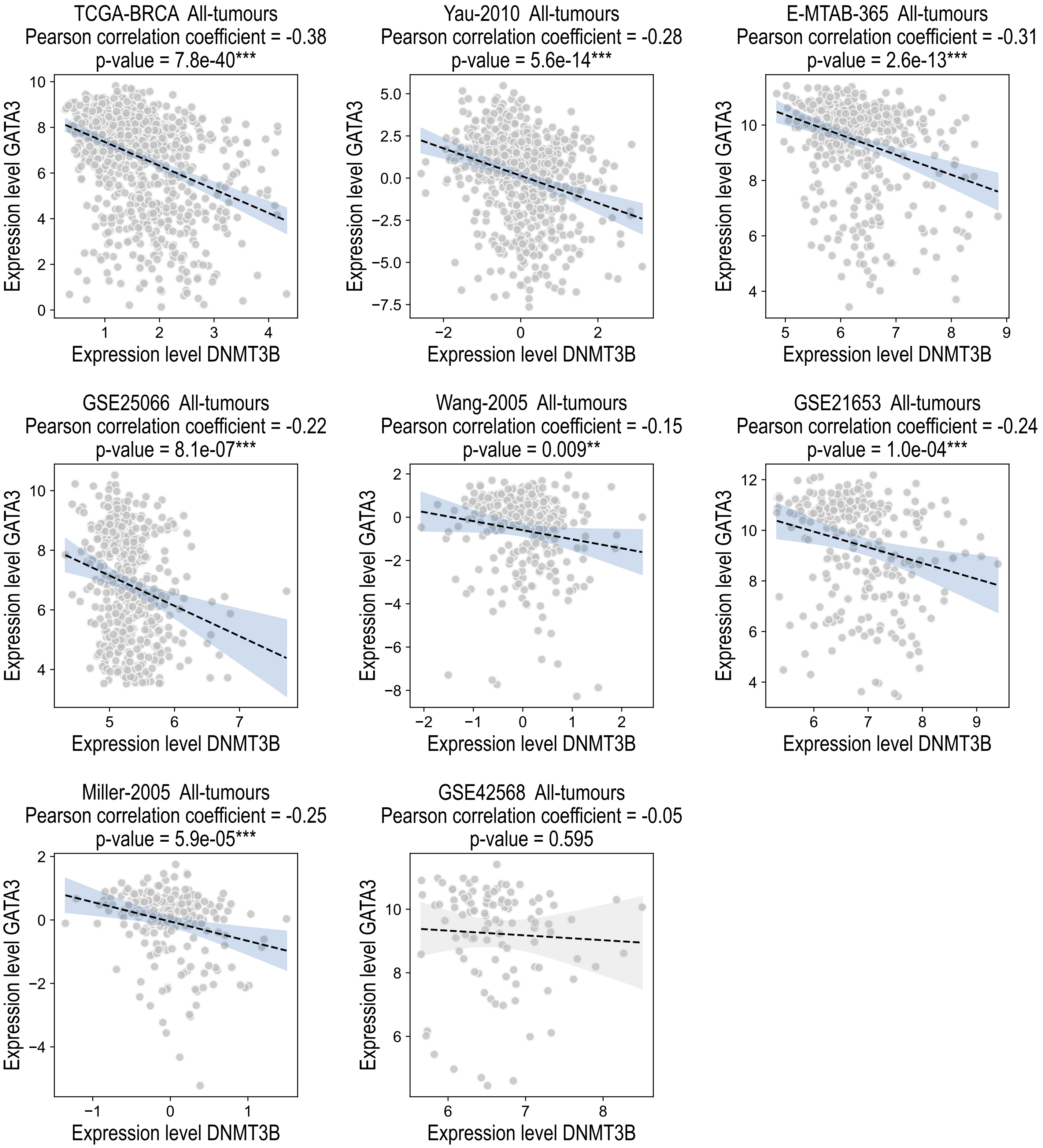

Supplementary Figure S1. Correlation plots between the expression levels of the genes *DNMT3B* and *GATA3* in eight public breast cancer datasets. The black dashed line shows the fitted regression. The shaded area corresponds to the 95% confidence interval in blue if the corresponding p-value < 0.05, otherwise in grey.
