# Supplementary material for: Discovery of epigenetically silenced tumour suppressor genes in aggressive breast cancer through a computational approach
Source: NAR Cancer. 2025 Jun 18;7(2):zcaf020. doi: 10.1093/narcan/zcaf020 (PMC12203794; doi:10.1093/narcan/zcaf020)
Supplement: zcaf020_Supplemental_Files [file zcaf020_supplemental_files.zip › Supplementary_Figure_S2.pdf]

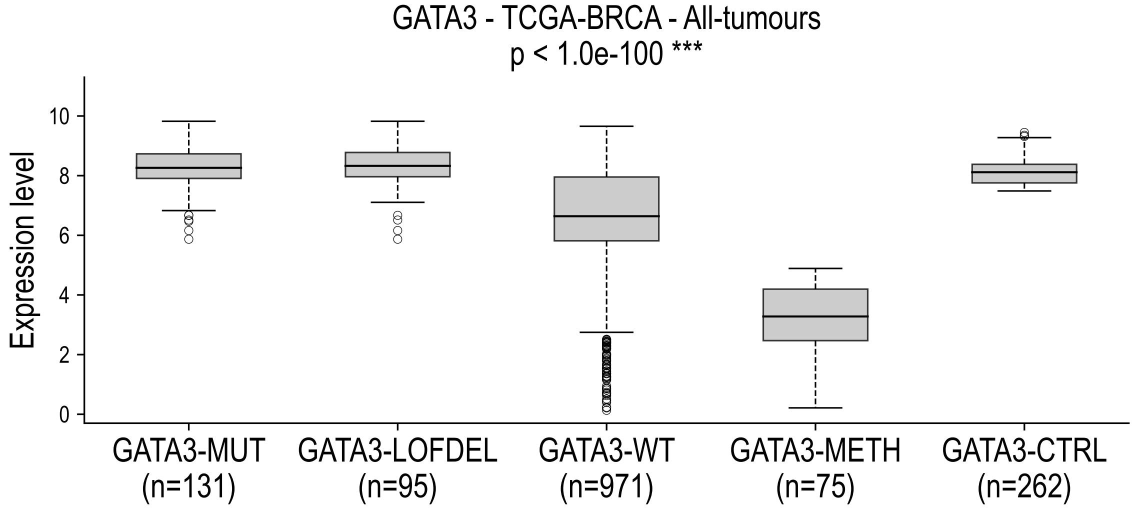

Supplementary Figure S2. Expression levels of the gene *GATA3* in five sample groups of the dataset TCGA-BRCA, categorized by *GATA3* mutation and/or promoter methylation status. GATA3-MUT: Samples with any type of *GATA3* mutation or deleterious CNA. GATA3-LOFDEL: Subset of GATA3-MUT samples carrying loss-of-function mutations. GATA3-WT: Wild-type *GATA3* samples, regardless of promoter methylation status. GATA3-METH: Samples with hypermethylation of the *GATA3* promoter region. GATA3-CTRL: Control group with neither *GATA3* mutations nor promoter hypermethylation.
